# Measuring Circularity in Food Supply Chain Using Life Cycle Assessment; Refining Oil from Olive Kernel

**DOI:** 10.3390/foods10030590

**Published:** 2021-03-11

**Authors:** Amin Nikkhah, Saeed Firouzi, Keyvan Dadaei, Sam Van Haute

**Affiliations:** 1 Department of Food Technology, Safety and Health, Faculty of Bioscience Engineering, Ghent University, Coupure Links 653, 9000 Ghent, Belgium; Sam.vanhaute@ghent.ac.kr; 2 Department of Environmental Technology, Food Technology and Molecular Biotechnology, Ghent University Global Campus, Incheon 21985, Korea; 3 Department of Agronomy, College of Agriculture, Rasht Branch, Islamic Azad University, Rasht 41476-54919, Iran; firoozi@iaurasht.ac.ir (S.F.); dadaee.keyvan@yahoo.com (K.D.)

**Keywords:** circular economy, environmental impact, global warming, valorization of waste

## Abstract

Valorization of food waste is a potential strategy toward a circular food supply chain. In this regard, measuring the circularity of food waste valorization systems is highly important to better understand multiple environmental impacts. Therefore, this study investigated the circularity of a food waste valorization system (refining oil from olive kernel) using a life cycle assessment methodology. An inventory of an industrial-based olive kernel oil production system is also provided in this study. The system boundary was the cradle to the factory gate of the production system. The results indicated that natural gas consumption was the highest contributor to most of the investigated impact categories. The global warming potential of one kg of oil produced from olive kernel was calculated to be 1.37 kg CO_2_eq. Moreover, the calculated damages of 1 kg oil production from olive kernel to human health, ecosystem quality, and resource depletion were 5.29 × 10^−7^ DALY, 0.12 PDF∙m^2^∙yr., and 24.40 MJ, respectively.

## 1. Introduction

The circular economy concept is gaining growing attention as an alternative to the linear economy—“take, make, waste,”—which exists now [1,2]. In a linear economy, natural resources are transformed into goods that provide economic value; however, they come with a limited life span, and are disposed of in the environment with minimum recovery of resources [3,4]. This system puts enormous stress on the carrying capacity of the planet [5]. The circular economy describes a system with minimum loss of resources by reusing, recycling, and recovering materials and energy [6,7,8,9]. Various strategies have been suggested for moving from a linear economy to a circular one, including R-based frameworks, such as the 3Rs strategy (reduce–reuse–recycle), the 4Rs (introducing “recover” as the fourth R), the 6Rs, and even the 9Rs [10,11].

The measurement of circularity is the first step in moving toward a circular system, as quoted by Peter Ducker: “what gets measured gets managed” [1]. There is not a unique approach for measuring a circular economy, since the understanding of a circular economy is still being explored [12]. To date, some assessment indices have been applied to measure circularity, such as the material circularity indicator [13], the circular economy index [14], material flow analysis [15], food loss and waste [16], and life cycle assessment (LCA) methodology [17,18]. Corona et al. (2019) [19] reviewed the applied approaches for measuring circularity and found three assessment frameworks, seven measurement indices, and nine assessment indicators. In this regard, LCA has been used for decades for the evaluation of the environmental impact of products and services but, more recently, it has shown to be a promising method to measure circularity. It is an appropriate method to investigate the environmental consequences of circular product designs and large-scale changes to move toward a more circular economy [8]. However, in recent years, LCA has been applied to measure circularity in various sectors, such as bio-based materials [20,21], tourism [5], and concrete production [22]. There are also some published documents addressing the connection between LCA and the circular economy concept in the food supply chain [16,23,24].

In the case of the food supply chain, approximately one-third of the total global food production, which is equal to 1.3 billion tons per year, is wasted in the food production/consumption chain [25]. This includes food loss (such as losses and spoilage at the producer level before the market) or waste (such as losses at retailer and consumer levels) [26]. In fact, food loss and waste (FLW) refer to a certain amount of food, nutrients, or calories that intentionally/unintentionally disappear from food systems [27]. A large part of FLW is avoidable, and could be decreased by implementing different strategies at each level of the life cycle of the production system [28,29]. Although food waste has been understood as a critical global issue [30], food waste has high potential for reuse or recovery in a circular economy prospective [31].

**Figure 1:**
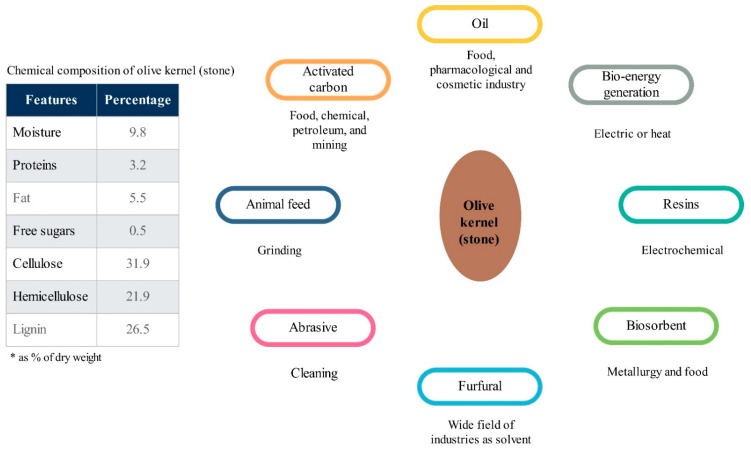
Characterization and application of olive kernel [37].

In this regard, the olive-based products industry is an interesting case, as it is an economically important industry [32]. As a globally energy-intensive sector, the olive processing industry faces sustainability challenges [33]. Espadas-Aldana et al. (2019) [34] studied 23 published papers on the LCA of olives and olive oil and concluded that the global warming potential (GWP) of one liter of olive oil production is equal to 1.6 kg CO_2_eq. The olive oil production supply chain also faces crucial challenges regarding waste management. For example, 80% of olive mass is composed of olive pulp and stones; therefore, waste production is four times higher than that produced within the extraction process [34]. In this regard, the by-products and residues generated in olive processing are not commonly used and end up as waste [33]. Thus, valorization of food waste could be considered as an effective strategy to make the supply chain of olive-based products more circular. In this regard, measuring the circularity of food waste valorization systems is highly important for improving understanding of multiple environmental impacts. One of these wastes is olive kernel (stone). Olive kernel is an important by-product generated in the pitted table olive industry [35]. The characterization and application of olive kernel are described in Figure 1. However, the current and main use of olive kernel is as direct solid feedstock for biofuel generation for domestic application [36]. However, this currently may not be a realistic option for an oil-rich country. In this regard, establishing an environmentally efficient approach for olive kernel utilization could actually improve the overall sustainability of olive-based product supply chains. This paper is the first report on the LCA of industrial-scale refining oil from olive kernel (as an olive processing waste valorization approach) system.

## 2. Materials and Methods

### 2.1. Refining Oil from Olive Kernel

**Figure 2:**
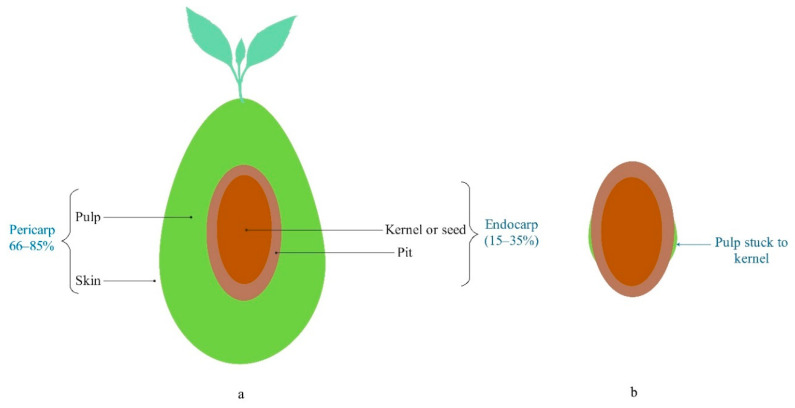
(**a**) different parts of an olive fruit [40,41], (**b**) the parts of the olive which were used to produce oil in the case study. Note: In this study, the term “kernel” refers to “kernel, pit, and pulp stuck to kernel”.

The olive kernel oil production company investigated in this study is located in Iran. Olives (*Olea europaea* L.) were grown in Iran, and the uses of olive fruits in the studied region are: (i) raw material for extra virgin olive oil production, (ii) inside dishes (pitted table olive), and (iii) raw material for pickling. Solid–liquid olive pomace and olive mill wastewater are the two major by-products of the extra virgin olive oil production system [38]. The kernel must be separated from the olive fruit in the second and third abovementioned olive fruit applications. Therefore, olive kernel is a common source of waste in olive fruit processing systems (Figure 2). Olive kernel can be used to produce oil. The characterization of olive kernel oil was described by Moghaddam et al. (2012) [39].

**Figure 3:**
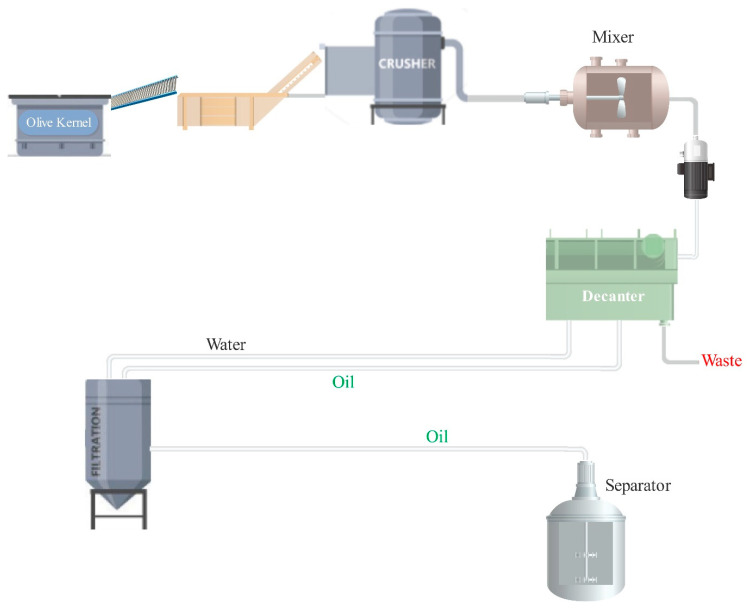
Olive oil production line with a daily production capacity of 100 kg.

The industrial olive kernel oil production process is shown in Figure 3. Olive kernels are transported to the processing plant, and the factory is located in the olive oil production/processing area. A small amount of olive pulp is stuck to kernels because it cannot be completely separated from the olive kernels in olive processing (Figure 2b). The received kernels are washed to remove impurities. Then, they are crushed to ease the release of the oil and are subsequently mixed. Afterward, the liquid (including oil and water) is separated through a decanter. In the next step, the oil is separated from the water and the olive kernel oil is extracted by a separator. Natural gas is consumed to heat the water in the boiler at a working temperature of 60 to 70 °C. Its circulation in the decanter’s double-walled jacket heats the dough (the crushed olive kernel, oil, and water). Mixer blades of the decanter provide a uniform spread of heat throughout the dough. Heating the dough contributes more efficiently to separating the three phases of oil, water, and pulp through centrifuging at 4000–4200 rpm. Moreover, the remaining pulp needs to be warmed to flow easily through the discharge mono pump of the decanter. Warm water is also added to the oil entering the separator in order to maximize the oil extraction rate. At the final step, a centrifuge rotating at a high rotational speed of 7000–7200 rpm separates the warm water and the olive kernel oil.

### 2.2. Measuring Circularity Using LCA

In this study, LCA methodology was applied to measure the circularity of an olive waste valorization system. Based on the review by Espadas-Aldana et al. (2019) [34] on olive oil LCA studies, the functional units (FUs) are defined as the quantity of olive oil produced in kg, and the energy content of the produced oil in MJ. Therefore, 1 kg of oil produced from olive kernel and 100 MJ energy generation were selected as the FUs for this LCA analysis to compare the environmental consequences associated with oil production from olive kernel with other vegetable-based oil production systems. The heating value (energy equivalent) of olive kernel oil was considered to be the same as for olive oil at 34.5 MJ/L [42]. The system boundary of this LCA study was the cradle-to-factory gate olive kernel oil production.

The cradle-to-gate environmental impact for olive kernel oil production was evaluated using LCA. In this regard, the main primary inventory data for olive kernel production in the considered factory are shown in Table 1. It should be noted that the input and output amounts were measured and recorded at the factory level, not through surveys or interviews. The experiments were performed in 2019. Experiments were conducted three times and the mean values are reported (Table 1). The olive kernel was not included as an input for the LCA analysis, and it was considered as a burden-free input; this is because it is a by-product/waste of a food production system. Therefore, the environmental impacts of wastewater from the olive kernel production system are not included in the system boundary. In other words, the assumption of not considering the wastewater treatment is due to the fact that olive kernel was not included as an input for the LCA analysis, as it is actually a by-product/waste of an olive processing system. Packaging is also excluded from the system boundary. The emitted pollutants were divided into off-site and on-site emissions. The emitted pollutants in the off-site phase (production of input materials) were adapted from the Ecoinvent 3.0 database using SimaPro 9.0.0.49 software. The datasets applied for calculation of emissions from off-site operations are shown in Appendix A, Table A1. The on-site emissions of natural gas consumption (see Appendix A, Table A2) were calculated and added to the on-site emission section using SimaPro software. The inventory of emissions for refining one kilogram oil from olive kernel is provided as Supplementary 1.

**Figure 4:**
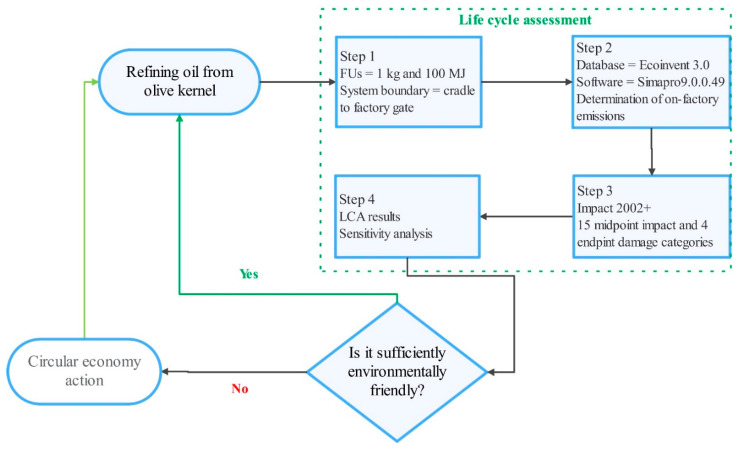
Measuring the circularity of olive kernel oil production using life cycle assessment (LCA) methodology.

In the third step of the LCA study, IMPACT 2002+ was employed as the impact assessment (IA) methodology due to its hybrid application of IMPACT 2002, Eco–Indicator 99, CML, and IPCC, all of which cover various impact and damage categories. This impact assessment (IA) methodology evaluates the environmental impacts based on the 15 impact categories, and also divides the impact categories into four damage categories: human health, ecosystem quality, GWP, and resource depletion. The human health damage category is represented as disability-adjusted life year (DALY), ecosystem quality as PDF∙m^2^∙yr., GWP as kg CO_2_eq, and resource depletion as MJ. The fourth and last step of conducting an LCA study is the interpretation of the LCA results, which are explained in the Results and Discussion section. The fourth step of an LCA study includes the determination of hotspots, the specification of areas with a potential for improvement, and recommendations [43]. A detailed explanation of this IA methodology can be found in Jolliet et al. (2003) [44]. A flowchart of the utilization of LCA for measuring the circularity of olive kernel oil production systems is demonstrated in Figure 4. Edraw Max (ver. 9.1, 2018; Sheung Wan, Hong Kong, China) software was used for the representation of graphical items.

There are several sources of uncertainty in an LCA study [45]. It is important to take into account the uncertainty of LCA results which can be due to a lack of accuracy in the collected data [46], the initial assumption [47], the allocation method [48], the selection of functional units [49], the determination of on-site emissions [50], and the type of IA methodology [51]. Therefore, to test the consistency of the LCA results obtained in this study, a quantitative uncertainty analysis was conducted to evaluate the effect of IA methodology selection on the final LCA results. In this regard, the GWP impact category was selected for comparison, as it is the mutual impact category among the investigated IA methodologies. The selected impact assessment methodologies were IMPACT 2002+, ReCiPe 2016 [52], CML–IA baseline [53], EDIP 2003 [54], EF [55], EN 15804 +A2 [56], Environmental Prices [57], EPD [58] and ILCD [59]. The IA methodologies evaluated by this study are available on SimaPro 9.0.0.49—a widely used software for LCA analysis [60,61].

One limitation of this research comes from the limited number (one industrial-scale company refining oil from olive kernel) of investigated olive kernel oil factories. Another limitation of this study is that, compared with studies on the LCA of extra virgin olive oils—the highest quality of olive oils—this olive kernel study does not consider the olive quality indices, as suggested by Salomone et al. (2015) [62].

## 3. Results and Discussion

### 3.1. Interpretation of Mid-Point LCA Results

The quantified amounts of the environmental impacts of olive kernel oil production, based on different impact categories, are presented in Table 2. The environmental impact of 1 kg of olive kernel oil production on GWP was calculated as 1.37 kg CO_2_eq. This value of the abovementioned impact category for 100 MJ energy generation was 4.32 kg CO_2_eq. The GWP of one liter of olive oil production is equal to 1.6 kg CO_2_eq, according to Espadas-Aldana et al. (2019) [34]. They also indicated that the agricultural phase is the most impactful phase in the olive oil supply chain, responsible for 0.46 kg CO_2_eq/kg of GWP. In the case of refining oil from olive kernel, olives are not used in the system, and the by-product of the olive postharvest waste was used to produce oil. Then, the environmental impacts of the agricultural phase are not accounted for in this system. Therefore, the environmental impacts of oil production from olive kernel are low, making the conventional olive processing systems more circular through waste valorization (olive kernel) of the system. If it is assumed that the production of oil from kernels means less olive oil needs to be produced elsewhere for cosmetic and pharmacological purposes (Figure 1), then this process can save around 0.23 kg CO_2_ per each kg of oil produced from olive kernel in the investigated system.

**Figure 5:**
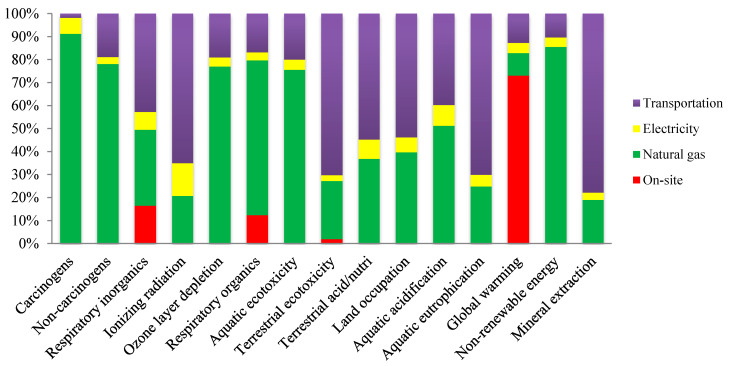
Contribution of consumed inputs to the environmental impact of the olive kernel oil production system.

Figure 5 demonstrates the proportion of inputs to the environmental effects of the olive kernel chain. Natural gas consumption was the highest contributor to the most investigated impact categories. The share of natural gas from off-farm emissions on GWP in olive kernel oil was 83%. On-site emissions contributed to the impact categories of GWP, respiratory organics, respiratory inorganics, and terrestrial ecotoxicity; contributions of on-site emissions to these impact categories were 73, 12, 16, and 2%, respectively.

### 3.2. Interpretation of End-Point Damage Assessment

**Figure 6:**
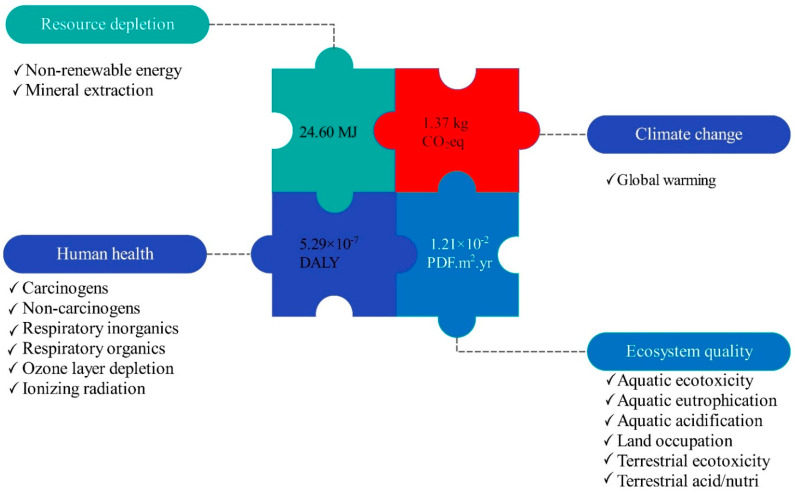
Damage assessment of olive kernel oil production (per 1 kg produced oil).

Figure 6 displays the damage assessment of the olive kernel oil chain. According to the results, the production of 1 kg of oil from olive kernel led to 5.29 × 10^−7^ DALY damage to human health. The current study investigated the environmental impacts associated with the olive kernel oil production system, and the chemical and microbiological health risk of the final produced oil was not involved in the LCA analysis. Further research is required to examine the human health risk caused by the consumption of oil produced from olive kernel, as well as to gain a proper understanding of the sustainability of the olive kernel oil production system throughout its life cycle. The damages of 1 kg of oil production from olive kernel to ecosystem quality, GWP, and resource depletion were 1.21 × 10^−2^ PDF·m^2^·yr., 1.37 kg CO_2_eq, and 24.40 MJ, respectively (Figure 6). The environmental indices of the investigated olive kernel oil production system based on different phases of the production system are shown in Table 3.

**Figure 7:**
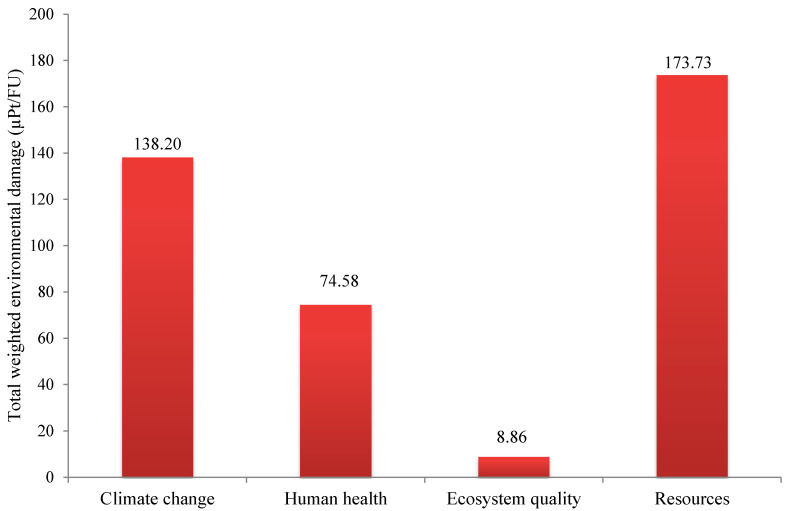
Single scores of damage categories in the olive kernel oil production chain (unit = µPt).

The single scores of the damage categories in olive kernel oil are shown in Figure 7. The total weighted environmental damage from refining oil from olive kernel was calculated as 395 µPt/FU. Natural gas was the largest contributor to the total environmental impacts of the studied system, with a share of 80% (including its background and on-site emissions). As explained in the Materials and Methods section, natural gas is used in olive kernel processing to heat water in order to (a) facilitate the separation of water and oil from pulp in the decanter, (b) contribute to the flow of the remaining pulp in the decanter, and (c) separate water from olive kernel oil in the separator. A preliminary experiment shows that the optimum dough temperature to extract purer oil is around 35 °C, which is associated with the circulating water temperature at around 60–70 °C in the studied system. Moreover, a temperature of around 35 °C for the water added to the separator might be efficient. Nevertheless, further research is needed to determine the optimum water temperature to avoid extra natural gas/heat consumption.

Khounani et al. (2021) [33] showed that the total environmental burdens of olive oil systems can be reduced by 12% by applying agro-biorefinery strategies based on olive cultivation and the extraction of fruit and pomace oil. There are also other ways to valorize olive processing wastes, such as energy generation. One LCA study highlighted significant greenhouse gas emission savings through olive husk (the solid portion remaining after pressing olives) application in a mobile pyrolysis process [63]. In another LCA study, Intini et al. (2012) [64] reported the environmental advantages of using de-oiled pomace and waste wood as feedstock for biofuel production, in terms of greenhouse gas emissions reduction. Multiple environmental measures could be applied in order to improve the sustainability of olive processing. In this regard, Martinez-Hernandez et al. (2014) [65] indicated how integrated process schemes can be used to develop a sustainable Jatropha-based biorefinery system.

### 3.3. Uncertainty of GWP’s Results

**Figure 8:**
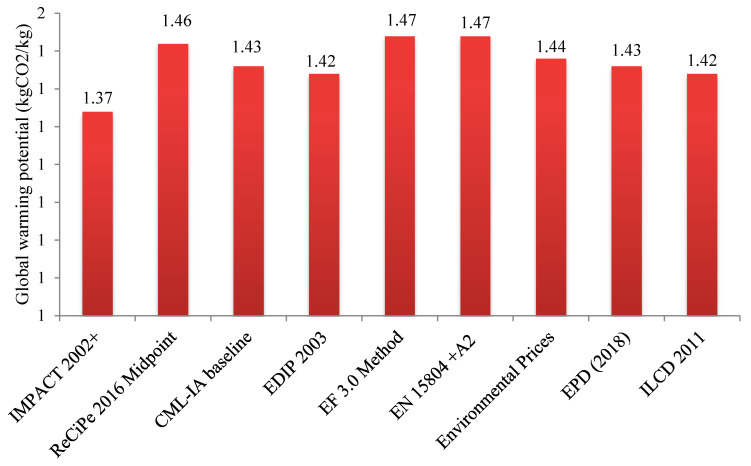
Effect of impact assessment methodology selection on the global warming potential (GWP) results.

A unique IA methodology for LCA analysis in food systems does not exist. Different IA methodologies may apply for characterization, and the selection of IA methodology can therefore affect the final LCA results. The characterization index of GWP for refining oil from olive kernel, based on the application of various IA methodologies, is illustrated in Figure 8. These results may correspond with the findings of relevant LCA studies on the same topic. The results revealed that the GWP of the production of 1 kg of olive kernel oil ranges from 1.37 to 1.47 kgCO_2_eq. The lowest estimation of GWP belonged to IMPACT 2002+, and the highest to the EDPI and EF methodologies. The results are in agreement with the reports by Fathollahi et al. (2018) [47] and Paramesh et al. (2018) [51], which indicated that selection of IA methodology can slightly affect LCA results in some impact categories in the food system. Therefore, it is recommended to consider the impact of IA selection as a source of uncertainty in future research concerning LCA in the food supply chain.

## 4. Conclusions

This study applied LCA to measure the circularity of a food waste system—in this case, refining oil from olive kernel. The damages of 1 kg of oil production from olive kernel to GWP, human health, ecosystem quality, and resource depletion were 1.37 kg CO_2_eq, 5.29 × 10^−7^ DALY, 1.21 × 10^−2^ PDF·m^2^·yr., and 24.40 MJ, respectively. The results highlighted that the studied system is relatively circular, resulting in low environmental impacts. Olive processing systems could be made more circular through food waste valorization. By managing the consumption of energy sources, such as natural gas, olive kernel oil production systems can become environmentally efficient. In this regard, further research is needed to determine the optimum temperature of olive kernel dough in the decanter, as well as the optimum temperature for warm water when added to the separator in the process of oil extraction from olive kernel. Further research is also required to explore the human health and microbiological risks of the oil produced from olive kernels, which were not considered in this study.

## Figures and Tables

**Table 1:** Main primary inventory data for the olive kernel oil production system.

Inputs and Outputs	Unit	Quantity	
		Per 1 kg Produced Oil	Per 100 MJ Produced Oil
Inputs			
—Olive kernel	kg	47.72	150.83
—Water consumption	m^3^	0.05	0.14
—Natural gas	m^3^	0.52	1.65
—Electricity	kWh	0.10	0.31
—Human labor	h	0.16	0.50
—Transportation of the produced oil	ton × km	0.34	1.07
Outputs			
—Olive kernel oil		1 kg	100 MJ
—Efficiency (oil/olive kernel)	%	2.09	2.09

**Table 2:** Environmental consequence of the olive kernel oil production system based on different impact categories.

Impact Category	Unit	Quantity	
		Per 1 kg Produced Oil	Per 100 MJ Produced Energy
Global warming	kg CO_2_eq	1.37	4.32
Non-renewable energy	MJ primary	26.40	83.44
Mineral extraction	MJ surplus	0.005	0.02
Ozone layer depletion	kg CFC-11eq	1.57 × 10^−7^	4.97 × 10^−7^
Non-carcinogens	kg C_2_H_3_Cl eq	0.02	0.05
Carcinogens	kg C_2_H_3_Cl eq	0.08	0.24
Ionizing radiation	Bq C-14 eq	1.94	6.14
Respiratory organics	kg C_2_H_4_ eq	3.84 × 10^−4^	1.39 × 10^−3^
Respiratory inorganics	kg PM2.5eq	4.40 × 10^−4^	1.21 × 10^−3^
Aquatic ecotoxicity	kg TEG water	68.07	215.16
Terrestrial ecotoxicity	kg TEG soil	12.71	40.16
Aquatic eutrophication	kg PO_4_ P-lim	3.28 × 10^−5^	1.04 × 10^−4^
Terrestrial acid/nutri	kg SO_2_eq	0.007	0.02
Land occupation	m^2^org.arable	0.009	0.03
Aquatic acidification	kg SO_2_eq	0.002	0.01

**Table 3:** Environmental impacts of the olive kernel oil production system based on different damage categories.

	Off-Site									On-Site		
	Electricity			Natural Gas			Transportation			On-Factory		
Damage Category	Damage Assessment	Normalized	Weighted	Damage Assessment	Normalized	Weighted	Damage Assessment	Normalized	Weighted	Damage Assessment	Normalized	Weighted
Human health	3.78 × 10^−8^ DALY	2.53 × 10^−6^	5.23	3.20 × 10^−7^ DALY	4.51 × 10^−5^	45.07	1.28 × 10^−7^ DALY	1.80 × 10^−5^	17.98	4.46 × 10^−8^ DALY	6.29 × 10^−6^	6.29
Ecosystem quality	3.95 × 10^−3^ PDF.m^2^.yr	2.8 × 10^−7^	0.29	3.46 × 10^−2^ PDF.m^2^.yr	2.53 × 10^−6^	2.53	8.08 × 10^−2^ PDF.m^2^.yr	5.89 × 10^−6^	5.89	2.02 × 10^−3^ PDF.m^2^.yr	1.47 × 10^−7^	0.15
Climate change	0.06 kg CO_2_eq	6.28 × 10^−6^	6.28	0.13 kg CO_2_eq	1.35 × 10^−5^	13.48	0.17 kg CO_2_eq	1.75 × 10^−5^	17.52	1.00 kg CO_2_eq	1.01 × 10^−4^	100.91
Resource depletion	1.08 MJ	7.12 × 10^−6^	7.12	22.57 MJ	1.49 × 10^−4^	148.52	2.75 MJ	1.81 × 10^−5^	18.09	0	0	0

## Data Availability

The inventory of the industrial-based olive kernel oil production system is provided in the paper. In addition, the inventory of emissions for refining one kilogram oil from olive kernel is also provided as Supplementary 1.

## Supplementary Materials

The following are available online at https://www.mdpi.com/2304-8158/10/3/590/s1, Supplementary 1.

## Appendix A

**Table A1:** Datasets applied for calculation of emissions from off-site operations.

Activity	Database	Category	Unit	Activity Uuid/Source
Electricity	Ecoinvent 3	IR	kWh	668baf12-38db-47b7-8517-c0a18aa122f4
Natural gas	Ecoinvent 3	GLO	M ^3^	65f221cf-b821-4da1-a2ed-a67994b10f42
Transport	Ecoinvent 3	GLO	t.km	413c356e-677d-4676-b816-0c0b20768d7a_03bf1369-1eec-49d0-bc4b-8b29efa826b9.spold
Water-Unspecified origins	Input from nature	IR	M ^3^	-

**Table A2:** Coefficients for calculation of emissions from natural gas combustion (gram).

Emission	Coefficients [66,67]	Quantity (per on kg Produced Olive Kernel Oil)
Carbon dioxide (CO_2_)	38.70	996.14
Methane (CH_4_)	7.40 × 10^−4^	1.90 × 10^−2^
Dinitrogen monoxide (N_2_O)	7.11 × 10^−4^	1.83 × 10^−2^
Sulfur dioxide (SO_2_)	1.93 × 10^−4^	4.97 × 10^−3^
Nickel (Ni)	6.78 × 10^−7^	1.75 × 10^−5^
Lead	1.61 × 10^−7^	4.14 × 10^−6^
Zinc (Zn)	9.37 × 10^−6^	2.41 × 10^−4^
Benzo(a)pyrene	3.87 × 10^−9^	9.96 × 10^−8^
Selenium (Se)	7.75 × 10^−9^	1.99 × 10^−7^
Organic compound	3.50 × 10^−3^	9.01 × 10^−2^
Volatile organic compound (VOC)	1.70 × 10^−3^	4.38 × 10^−2^
Particulates (<2.5 μm)	2.45 × 10^−3^	6.31 × 10^−2^
